# Sex-specific developmental models for *Creophilus maxillosus* (L.) (Coleoptera: Staphylinidae): searching for larger accuracy of insect age estimates

**DOI:** 10.1007/s00414-017-1713-4

**Published:** 2017-10-24

**Authors:** Katarzyna Frątczak-Łagiewska, Szymon Matuszewski

**Affiliations:** 1 Laboratory of Criminalistics, Adam Mickiewicz University, Św. Marcin 90, 61-809 Poznań, Poland; 2 Department of Animal Taxonomy and Ecology, Adam Mickiewicz University, Umultowska 89, 61-614 Poznań, Poland

**Keywords:** Forensic entomology, Postmortem interval, Development, Age estimates, Insect sex, Estimation accuracy

## Abstract

Differences in size between males and females, called the sexual size dimorphism, are common in insects. These differences may be followed by differences in the duration of development. Accordingly, it is believed that insect sex may be used to increase the accuracy of insect age estimates in forensic entomology. Here, the sex-specific differences in the development of *Creophilus maxillosus* were studied at seven constant temperatures. We have also created separate developmental models for males and females of *C. maxillosus* and tested them in a validation study to answer a question whether sex-specific developmental models improve the accuracy of insect age estimates. Results demonstrate that males of *C. maxillosus* developed significantly longer than females. The sex-specific and general models for the total immature development had the same optimal temperature range and similar developmental threshold but different thermal constant *K*, which was the largest in the case of the male-specific model and the smallest in the case of the female-specific model. Despite these differences, validation study revealed just minimal and statistically insignificant differences in the accuracy of age estimates using sex-specific and general thermal summation models. This finding indicates that in spite of statistically significant differences in the duration of immature development between females and males of *C. maxillosus*, there is no increase in the accuracy of insect age estimates while using the sex-specific thermal summation models compared to the general model. Accordingly, this study does not support the use of sex-specific developmental data for the estimation of insect age in forensic entomology.

**Electronic supplementary material:**

The online version of this article (10.1007/s00414-017-1713-4) contains supplementary material, which is available to authorized users.

## Introduction

One of the entomological methods for postmortem interval (PMI) estimation is the developmental method. It involves estimating the age of the oldest immature stages of insects found on a cadaver [1–3]. For this purpose, indicators of insect age, such as larval length or weight, are measured. Observed values of indicators are then compared with developmental data included in a species-specific developmental model [4, 5]. The whole procedure requires access to case-specific temperature data [6, 7]. Developmental models are created in laboratory experiments where insects are usually kept in constant temperatures and measured frequently [8]. Their results are presented using graphical (e.g., isomegalen and isomorphen diagram) or mathematical models [3, 4]. In practice, age of insects is usually estimated using linear models (i.e., thermal summation models) [3, 9]; however, recently forensic entomologists have become more interested in nonlinear models [10, 11].

The variety of factors affects accuracy of the development-based PMI estimates. Many of them influence directly the accuracy with which insect age is estimated, and the quality of the developmental model is one of the highest importance [12, 13]. Developmental models should be based on experiments carried out in at least six temperatures, age indicators should be measured at intervals representing no more than 10% of the stage duration, and the median should be the statistic used to characterize duration of development [12]. Apart from these factors, accuracy of insect age estimation depends on the appropriateness of the model used (i.e., the degree of match between the conditions under which the model was developed and the conditions under which insects from this particular cadaver have been developing) as well as the quality of temperature data used [6, 7]. Nonetheless, even a very accurate estimate of insect age indicates just the minimum PMI, which may substantially differ from the actual PMI. Therefore, apart from the factors indicated above, the accuracy of PMI estimation using the developmental method may depend on factors affecting length of the period preceding appearance of insects on a cadaver (i.e., the pre-appearance interval, PAI) [14]. In the case of insects colonizing cadavers shortly after death (e.g., blowflies), their age is usually very close to the actual PMI. However, in the case of insects colonizing carcasses later in decomposition, it is usually necessary to estimate PAI in some way, for example, with the use of the temperature methods [14]. Additionally, it should be evaluated how accurately the entomofauna of a cadaver is represented by the insect sample (in particular, whether the oldest insects were sampled) and what factors may have affected colonization of a body by insects in this particular case. In cases of negligence, a body may be colonized before death [15], whereas in a burial scenario [16], after wrapping in material [17], in an indoor scenario [18, 19], in a car [19], or in a suitcase [20], colonization by insects may be delayed. The same effect may result from the bad weather [21], or a night-time cadaver exposure [22, 23].

In forensic entomology, efforts are now being made to improve the accuracy of insect age estimation through, among others, improving the quality of developmental models. From this point of view, insect sex has recently focused attention of researchers [11, 24]. Usually, sex may easily be identified in the adult stage. Accordingly, sex of immature insects collected from the cadaver may be determined after breeding them to the adult stage in the laboratory. There are also molecular methods of sex determination [25]. There are widespread differences in size between males and females of insects, called the sexual size dimorphism [26–29]. They may result from differences between sexes in size at hatching, rate of development, length of development, or any combination of these factors [30, 31]. Accordingly, sex-specific differences in length of development have been found in many insect species [27, 28], including species used in forensic entomology [11, 24]. It can therefore be assumed that such differences may occur in many other species of forensically important insects. Sexual size dimorphism is present in many species of necrophilous beetles, e.g., *Necrodes littoralis* (Linnaeus, 1758), *Creophilus maxillosus* [32], *Dermestes maculatus* (DeGeer, 1774) [33, 34], and flies, e.g., *Chrysomya megacephala* (Fabricius, 1794) [35] or *Megaselia scalaris* Loew, 1866 [11]. We believe that these differences in size are followed by differences in the duration of development. It is however unclear whether these differences may increase the accuracy of age estimates while using sex-specific models of development. Although some researchers suggested that assessment of sex-specific growth may reduce noise in minimum PMI estimates [24], no previous study validated sex-specific developmental data or created sex-specific developmental models for PMI estimation.

*C. maxillosus* is a predatory beetle that feeds mainly on larvae of necrophagous flies [36–38]. It regularly visits large vertebrate cadavers in natural (non-urban) environments [39–42]. Moreover, due to its large size, it may be easily sampled during cadaver inspection. It colonizes cadavers much later than most flies, so its use may substantially prolong the period when PMI is estimated using the developmental method [37, 43]. Additionally, PAI of *C. maxillosus* may easily be estimated using temperature methods [44]. It is therefore a perfect species for the PMI estimation based on the combination of the developmental method and the PAI method. Although *C. maxillosus* was regularly sampled from human cadavers, it was rarely used to estimate PMI (mostly with succession-based approach) [39, 45]. Its infrequent use resulted from lack of developmental data for this species and colonization of cadavers exclusively in the natural habitats.

The aim of this study was to test whether there are differences in development time between sexes of *C. maxillosus* and at what stage of development they appear. Moreover, we created separate developmental models for males and females, and made validation studies to test whether such models improve the accuracy of age estimation. The following predictions were formulated: (1) Males of *C. maxillosus* develop longer than females. (2) Sex-specific differences in development time accumulate across all stages; however, they are the largest at the third larval and pupal stages. (3) The use of sex-specific developmental models substantially improves the accuracy of insect age estimates and consequently minimum PMI.

## Materials and methods

### Maintaining *C. maxillosus* colony in the laboratory

A laboratory colony was established twice, in 2015 and 2016. In each year, about 50 adult beetles were collected manually from rabbit carcasses placed in a xerothermic grassland in the Biedrusko military range (Western Poland, Europe; 52 31′ N, 16 55′ E) during spring and summer. All the time, the colony consisted of 25–30 individuals (more or less equal ratio of males and females). New beetles sampled from the field carcasses and individuals bred in the laboratory were added to the colony. Insects were kept in plastic containers (30.4 × 20 × 20.1 cm) and were fed once a day with blowfly pupae or third instar larvae. Moist soil (6–7 cm) was used, and containers were cleaned every 6–8 days to avoid appearance of mites and mold. Insects were kept at room temperature and humidity (20–22 °C, 50–60%).

### Laboratory protocol

Development was studied at seven constant temperatures: 15, 17.5, 20, 22.5, 25, 27.5, and 30 °C. In order to get eggs, all adult insects from the colony were put into a 3-l container filled halfway with soil (temperature 20–22 °C). After 4 h, adult beetles were pulled out and containers were placed in insect incubators (ST 1/1 BASIC or +, POL-EKO, Poland) with the predefined temperature. After 70% of the average egg stage duration, containers were inspected for the presence of first instar larvae, at intervals equal to 10% of the average egg stage duration. We used such methods, as *C. maxillosus* lay singular eggs in small clumps of soil which makes them very difficult to be found. Freshly hatched first instar larvae are very active and creamy-white in color, so it is not possible to omit them while searching the soil. Only freshly hatched larvae were sampled and transferred to separate cups, each larva to a single cup. Forty larvae per temperature were used. Immature beetles came from separate ovipositions (laid by different females originating from the highly variable colony). Two or three temperatures were studied at the same time, and insects were randomly allocated to temperatures. First and second instar larvae were kept in 80-ml containers with 1.5 cm of soil, third instar larvae and pupae in 120-ml containers with 5 cm of soil. Larvae were fed once a day with third instar larvae of blowflies killed and punctured to make feeding easier for the first and second instar *C. maxillosus*. Humidity in insect incubators was maintained at 60–70%, and a photoperiod (h) was set on 12:12 (L/D).

Five developmental landmarks were defined: hatching, first ecdysis, second ecdysis, pupation, and adult emergence. All individuals were inspected for developmental landmarks; half of them (chosen at random) were also repeatedly measured and weighed. After a landmark had been recorded, the midpoint between the current and the previous inspection was used as the actual time of the landmark occurrence. Transitions between larval stages were determined based on the color of a larva (creamy-white shortly after ecdysis) and the width of the mesonotum [46]. Sex of beetles was determined after emergence on the basis of the shape of the eighth abdominal sternite.

### Inspections and measurements of larvae and pupae

Inspections and measurements were carried out at intervals representing 10% of the life stage duration [12] (Table 1). A geometrical micrometer was used to measure in vivo larval length [47]. A larva was placed in a 1.5-ml Eppendorf tube, and after it had become immobile and fully erected, its length (from clypeus to the last abdominal segment) was measured with a micrometer. An analytical balance AS 82/220.R2 (Radwag, Poland) was used to weigh larvae and pupae while being kept in a 1.5-ml Eppendorf tube.

**Table 1:** Inspection/measurement intervals (hours) in each temperature

Developmental stage	Temperature (°C)						
	15	17.5	20	22.5	25	27.5	30
Egg	12	12	7	7	5	5	4
1st instar larva	9	9	6	6	4	4	3
2nd instar larva	11	11	8	8	6	6	5
3rd instar larva	40	40	30	30	24	24	18
Pupa	30	30	24	24	16	16	12

### Statistical analyses

Differences between males and females in the duration of developmental stages and total immature development as well as in the adult insect length and weight at emergence were evaluated using the *t* test for independent samples. Thermal summation models for the total immature development were calculated separately for males and females, as well as for the pooled sample (i.e., the general model), using the equation proposed by Ikemoto and Takai [9]. Eight insects per temperature and sex were randomly selected to be used for the modeling purposes; the rest of the specimens were used to test performance of the models in the age estimation task. Due to the large mortality at extreme temperatures, 15 and 30 °C were poorly represented in the validation sample. The validation included comparison of the thermal units needed to reach the adult stage, as calculated for each specimen at relevant developmental threshold, with the thermal constant from the model. The accuracy with which the model represented the actual thermal units needed for the emergence of the adult stage was compared across models using the *t* test for correlated samples. All analyses were performed using Statistica 12 (StatSoft, Inc., 2014) at 5% level of significance.

## Results

### Differences in development between sexes of *C. maxillosus*

**Fig. 1:**
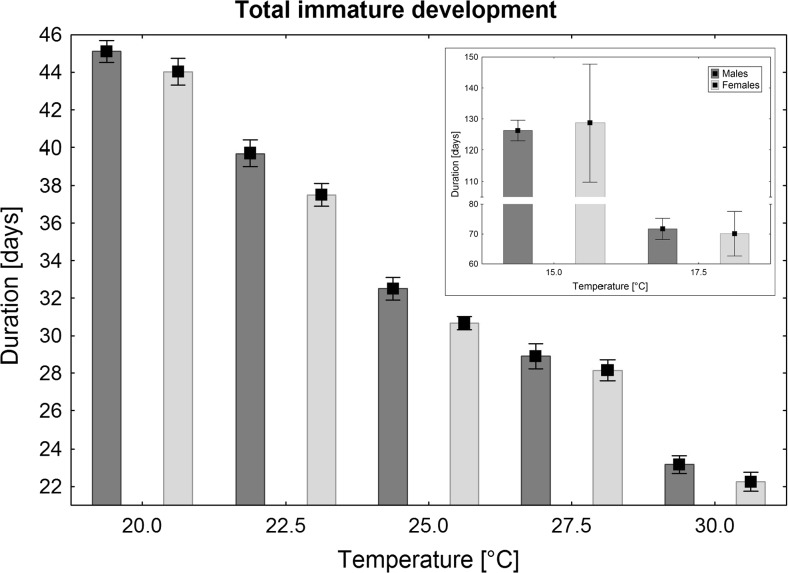
Differences between males and females of *C. maxillosus* in duration of total immature development at seven constant temperatures; black box—mean, whiskers—standard error of the mean

**Fig. 2:**
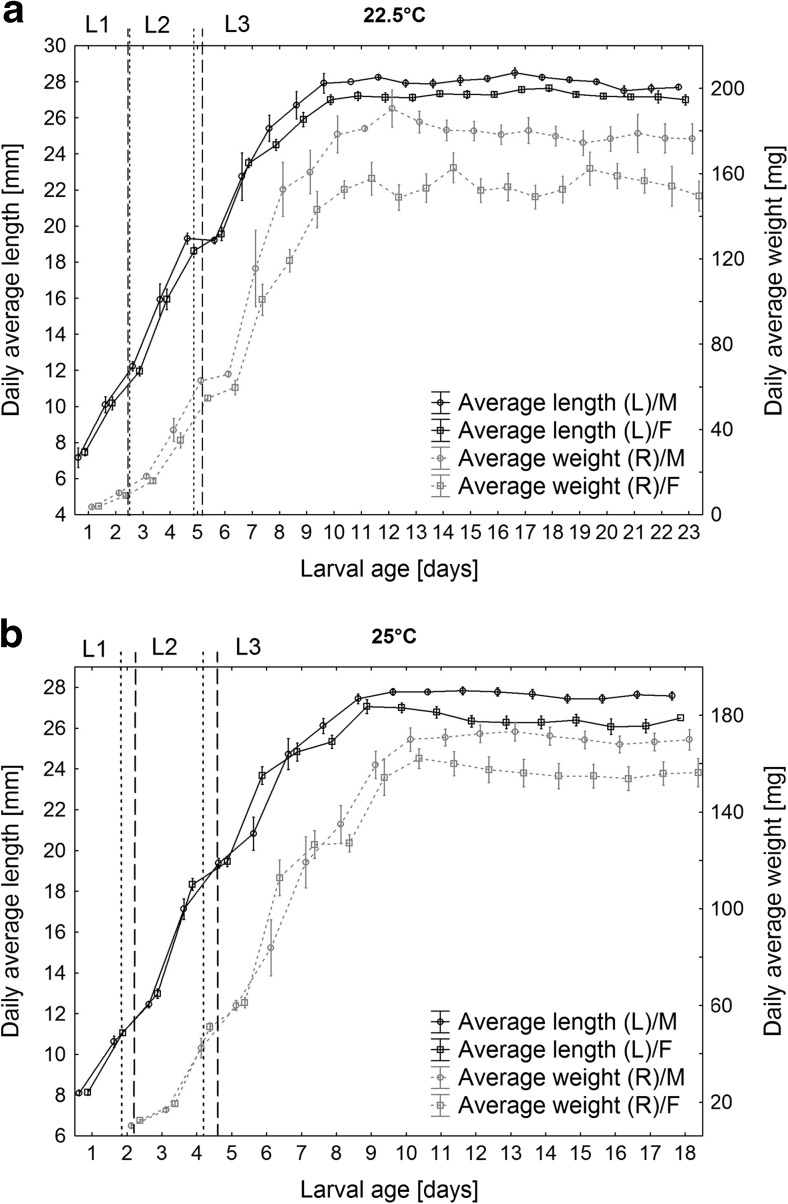
Growth curves for males and females of larval *C. maxillosus* at constant temperatures of 22.5 °C (**a**) and 25 °C (**b**); symbols—mean, whiskers—standard error of the mean; L1—first larval stage, L2—second larval stage, L3—third larval stage; dotted lines indicate the average moment of transition to the next stage for females (- - - - ) and males (— —); M-males, F-females, L-left *y* axis, R-right *y* axis. Daily average length and weight are average values calculated across all measurements of the given larva in a given day

As a rule, males of *C. maxillosus* developed longer than females (Fig. 1, Table 2, electronic supplementary material). Differences in the duration of development between females and males were largest at 22.5 and 25 °C and in the third larval and pupal stages (Fig. 1, Table 2, electronic supplementary material). The differences were statistically insignificant in most single-stage comparisons, partly due to their small size and partly due to the small size of samples used in the comparisons (Table 2). Because the differences have accumulated over the entire premature development, at eclosion they were quite large and in the case of 22.5 and 25 °C statistically significant, for example, at 25 °C males of *C. maxillosus* emerged on average almost 2 days later than females (Table 2). Males were distinctly larger (longer and heavier) than females from the beginning of the third larval stage until eclosion (Fig. 2). After eclosion, adult males were about 1.5 mm longer and about 20 mg heavier than adult females; however, at higher temperatures, starting from 25 °C, these differences were smaller (Table 3).

**Table 2:** Differences in the duration of development between males and females of *C. maxillosus* at different temperatures and for different stages

Temperature (°C)	Developmental stage	Mean duration (days) (SD; *N*)		*t*	*P*
		Females	Males		
15	Egg	8.36 (0.13; 3)	8.53 (0.35; 11)	− 0.81	0.43
	1st instar larva	4.95 (0.24; 3)	5.23 (1.01; 11)	− 0.45	0.66
	2nd instar larva	5.00 (0.46; 3)	5.38 (0.40; 11)	− 1.40	0.19
	3rd instar larva	85.23 (29.46; 2)	81.06 (8.46; 10)	0.44	0.67
	Pupa	23.54 (3.15; 3)	26.23 (2.55; 11)	− 1.55	0.15
	Total	128.75 (26.81; 2)	126.24 (10.58; 10)	0.25	0.81
17.5	Egg	5.94 (0.51; 6)	5.86 (0.41; 15)	0.35	0.73
	1st instar larva	4.26 (0.70; 6)	4.21 (0.60; 15)	0.14	0.89
	2nd instar larva	4.13 (0.31; 6)	4.25 (0.28; 15)	− 0.90	0.38
	3rd instar larva	37.44 (17.09; 6)	38.17 (13.87; 15)	− 0.10	0.92
	Pupa	18.33 (0.68; 6)	19.21 (1.27; 15)	− 1.58	0.13
	Total	70.10 (18.23; 6)	71.71 (13.67; 15)	− 0.22	0.83
20	Egg	4.31 (0.13; 16)	4.30 (0.16; 16)	0.30	0.76
	1st instar larva	2.74 (0.22; 16)	2.79 (0.27; 16)	− 0.51	0.61
	2nd instar larva	3.12 (0.21; 16)	3.06 (0.26; 16)	0.74	0.47
	3rd instar larva	18.29 (2.86; 16)	19.15 (1.75; 16)	− 1.02	0.31
	Pupa	15.56 (0.63; 16)	15.81 (0.97; 16)	− 0.88	0.39
	Total	44.04 (2.88; 16)	45.11 (2.34; 16)	− 1.16	0.25
22.5	Egg	3.34 (0.11; 17)	3.37 (0.21; 13)	− 0.41	0.69
	1st instar larva	2.26 (0.22; 17)	2.37 (0.35; 13)	− 1.05	0.30
	2nd instar larva	2.55 (0.19; 17)	2.47 (0.29; 13)	0.86	0.40
	3rd instar larva	17.26 (2.43; 17)	19.06 (2.68; 13)	− 1.92	0.06
	Pupa	12.10 (0.80; 17)	12.43 (0.48; 13)	− 1.34	0.19
	Total	37.51 (2.52; 17)	39.70 (2.50; 13)	− 2.37	0.02
25	Egg	2.80 (0.09; 22)	2.86 (0.10; 14)	− 1.76	0.09
	1st instar larva	1.90 (0.15; 22)	2.00 (0.37; 14)	− 1.09	0.28
	2nd instar larva	2.24 (0.16; 22)	2.40 (0.14; 14)	− 3.09	0.004
	3rd instar larva	14.12 (1.50; 22)	14.98 (1.86; 14)	− 1.52	0.14
	Pupa	9.60 (0.63; 22)	10.26 (0.51; 14)	− 3.32	0.002
	Total	30.67 (1.62; 22)	32.51 (2.28; 14)	− 2.84	0.008
27.5	Egg	2.57 (0.14; 15)	2.60 (0.13; 12)	− 0.74	0.47
	1st instar larva	1.70 (0.30; 15)	1.72 (0.24; 12)	− 0.16	0.88
	2nd instar larva	2.21 (0.31; 15)	2.21 (0.26; 12)	< 0.01	0.99
	3rd instar larva	13.56 (1.91; 15)	13.79 (1.75; 12)	− 0.33	0.75
	Pupa	8.58 (0.78; 15)	8.12 (1.13; 12)	− 1.26	0.22
	Total	28.15 (2.13; 15)	28.9 (2.34; 12)	− 0.87	0.39
30	Egg	2.20 (0.15; 10)	2.21 (0.07; 3)	− 0.05	0.96
	1st instar larva	1.41 (0.14; 10)	1.52 (0; 3)	− 1.37	0.20
	2nd instar larva	1.69 (0.14; 10)	1.83 (0; 3)	− 1.74	0.11
	3rd instar larva	9.63 (1.65; 10)	9.98 (0.43; 3)	− 0.35	0.73
	Pupa	7.32 (0.42;10)	7.62 (0.87; 3)	− 0.86	0.41
	Total	22.25 (1.61;10)	23.17 (0.81; 3)	− 0.92	0.37

**Table 3:** Differences between males and females of *C. maxillosus* in length and weight at emergence

Insect size	Temperature (°C)	Mean (SD; *N*)		*t*	*P*
		Females	Males		
Length (mm)	15	18.2 (2.1; 3)	20.7 (1.6; 11)	− 2.32	0.04
	17.5	19.7 (1.0; 6)	21.3 (1.3; 15)	− 2.76	0.01
	20	21.1 (1.2; 16)	22.3 (1.4; 16)	− 2.53	0.02
	22.5	19.7 (1.5; 17)	21.1 (1.6; 13)	− 2.43	0.02
	25	17.7 (1.0; 22)	18.6 (0.8; 14)	− 2.81	0.008
	27.5	17.0 (1.3; 15)	18.5 (1.7; 12)	− 2.65	0.01
	30	17.8 (1.4; 10)	17.8 (0.8; 3)	0.02	0.98
Weight (mg)	15	142.5 (23.6; 3)	160.6 (25.1; 11)	− 1.12	0.29
	17.5	136.6 (13.9; 6)	158.0 (16.6; 15)	− 2.77	0.01
	20	146.5 (17.3; 16)	166.4 (28.4; 16)	− 2.40	0.02
	22.5	127.8 (14.7; 17)	149.7 (20.0; 13)	− 3.46	0.002
	25	120.1 (10.4; 22)	130.2 (8.5; 14)	− 3.05	0.004
	27.5	107.1 (16.4; 15)	117.7 (23.4; 12)	− 1.38	0.18
	30	97.3 (10.1; 10)	98.9 (10.9; 3)	− 0.23	0.82

### Sex-specific developmental models of *C. maxillosus*

**Fig. 3:**
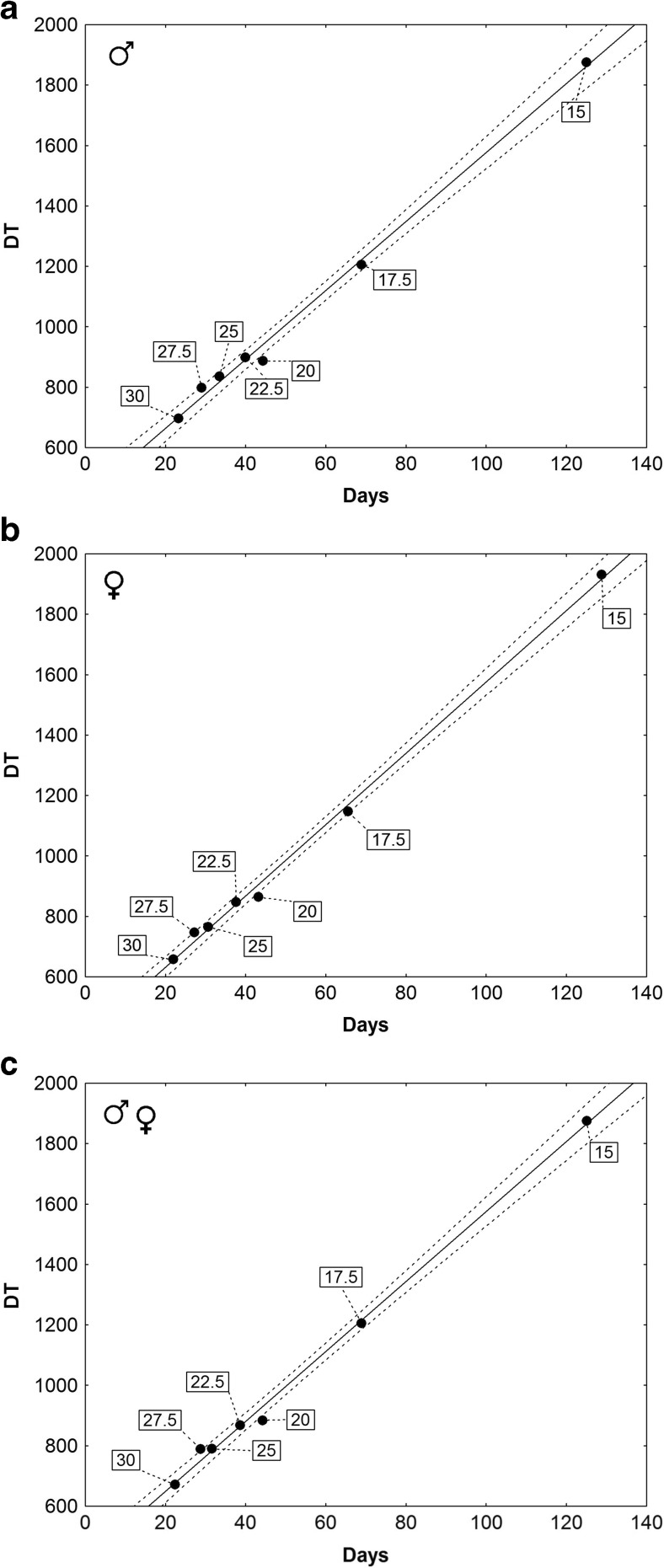
Thermal summation models for the total immature development of *C. maxillosus* males (**a**), females (**b**), and pooled sample (**c**); DT is the time in days to reach the adult stage multiplied by the constant rearing temperature

**Fig. 4:**
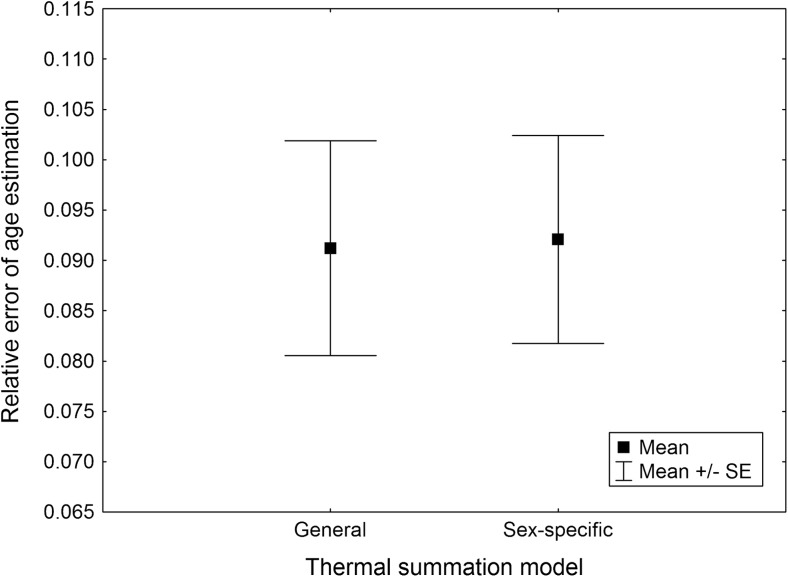
Relative error in age estimation of *C. maxillosus* at emergence using sex-specific (for males and females) and general models (for pooled sample)

All temperature points were included while calculating model parameters (Fig. 3). The models have the same optimal temperature range, similar developmental threshold, and different thermal constant *K*, which was the largest in the case of the male-specific model and the smallest in the case of the female-specific model (Table 4). Despite these differences, validation study revealed just minimal and statistically insignificant differences in the accuracy of age estimates using sex-specific and general models (*t* test for correlated samples; *t* = − 0.25, *P* = 0.80, Fig. 4).

**Table 4:** Thermal summation models for the total immature development of *C. maxillosus*

Model	Temperature range (°C)	Thermal summation constant—*K* (SE) (days °C)	Developmental threshold—*D*_0_ (SE) (°C)	*r* ^*2*^	*N*	*P*
Sex-specific (males)	15–30	434.25 (22.06)	11.43 (0.36)	0.995	7	< 0.001
Sex-specific (females)	15–30	395.27 (17.83)	11.81 (0.29)	0.997	7	< 0.001
General (males and females)	15–30	417.33 (19.52)	11.58 (0.32)	0.996	7	< 0.001

## Discussion

### Differences in development between sexes of *C. maxillosus*

Differences in duration of development between sexes were in line with our expectations. Final size of an insect should be proportional to the duration of growth [48]. As *C. maxillosus* males are larger than females, we have correctly assumed that they will develop longer. Sexual differences in the duration of development have already been studied in necrophagous fly species *Lucilia sericata* (Meigen, 1826) [24] and *M. scalaris* [11]. In both species, females are larger and thus develop longer than males. This pattern is much more common among various insect species [28, 31], including forensically important ones, e.g. *D. maculatus* [33, 34] or *C. megacephala* [35]. Large females gain more than males in terms of fitness [49] as they can lay more eggs of good quality [50]. In the case of males, large body size may be crucial while competing for limited resources, i.e., food or mating candidates. Larger males are common among predacious species (e.g., Staphylinidae) probably because of the high level of intra-sexual competition for limited resources [32]. All these studies suggest that sexual differences in development time may be prevalent in forensically important insects, as many of them are characterized by the sexual size dimorphism, e.g., *N. littoralis* [32].

The developmental differences between sexes were not large, but at eclosion they were already substantial due to their accumulation during development. The differences were the largest for the third larval and pupal stages, as these life stages are the longest in the case of *C. maxillosus* [37, 38]. In the case of *M. scalaris*, differences in development between males and females were the largest in the pupal stage [11]. For many fly species, the pupal stage is the longest life stage, frequently representing about 50% of the total immature development [51, 52]. For many beetle species, the longest life stage is the third larval stage [53, 54]. Consequently, it is likely that sexual differences in duration of development will be the largest in the third larval stage in the case of beetles and in the pupal stage in the case of flies.

### Sex-specific developmental models of *C. maxillosus*

We expected that the use of sex-specific developmental models will substantially improve the accuracy of insect age estimates. Despite significant differences in the duration of development between males and females, the improvement has not been achieved. The differences in duration of development are probably too small to have consequences for the accuracy of age estimates using sex-specific developmental models. This finding draws attention to a very important issue in the case of any new technique in forensic science, that is, its validation. Current results demonstrate that statistically significant effect is not always equivalent to practically relevant effect, which has recently been highlighted by Wells and LaMotte [55].

Sexual differences in the duration of particular life stages were small. Consequently, it is not worth creating sex-specific developmental models for particular stages, possibly just for the entire development. In this study, the largest differences between males and females occurred at 22.5 and 25 °C. In both temperatures, females completed development in about 95% of the time required by males. Similar differences in the duration of development between sexes were reported for *M. scalaris* [11] and *L. sericata* [24], except that females developed longer than males. In the case of *M. scalaris*, males completed development in 92.5% of the time required by females [11], and in the case of *L. sericata*, males completed development in 94.5% of that time [24]. Although these authors either did not create sex-specific developmental models or did not validate them, the differences reported are similar to the current differences. Therefore, it is probable that in the case of *L. sericata* and *M. scalaris* sexual differences in developmental time are, similarly, too small to improve the accuracy of insect age estimates.

Because our results do not support the use of sex-specific developmental data in forensic entomology, it would be useful to test the effect the sex-specific developmental models may have on the accuracy of age estimates in the case of insect species with larger size differences between males and females. Moreover, other techniques to improve the accuracy of insect age estimates in forensic entomology are necessary. Because insect size is highly intra-sexually variable, the better solution may be to use the size of an insect instead of its sex.

## Self-critique

### Fewer individuals at extreme temperatures

In low and high temperatures, the development of fewer individuals has been analyzed due to the high mortality. Moreover, females and males survived differently in different temperatures, with more females surviving in high temperatures and more males in low temperatures. Although we had enough data from all temperatures to create sex-specific models, extreme temperatures were underrepresented in the validation part of the study.

### Study of development at constant temperatures

Although insects develop in natural environment at fluctuating temperatures, the study was made at constant temperatures to enable comparison with results of other studies (development of forensically useful insects is usually studied under constant temperature) and make it possible to create accurate thermal summation models avoiding rate summation effect [3].

## Electronic supplementary material

ESM 1 (PDF 567 kb)
