# Supplementary material for: Sex-specific developmental models for Creophilus maxillosus (L.) (Coleoptera: Staphylinidae): searching for larger accuracy of insect age estimates
Source: Int J Legal Med. 2017 Oct 24;132(3):887–95. doi: 10.1007/s00414-017-1713-4 (PMC5919984; doi:10.1007/s00414-017-1713-4)
Supplement: Supplementary file 1 [file 414_2017_1713_MOESM1_ESM.pdf]

SUPPLEMENTARY FIGURES FOR

"Sex-specific developmental models for *Creophilus maxillosus* (L.) (Coleoptera: Staphylinidae): searching for larger accuracy of insect age estimates"

International Journal of Legal Medicine

Katarzyna Frątczak-Łagiewska M.Sc.[1,2*], Szymon Matuszewski Ph.D.[1]

[1]Laboratory of Criminalistics, Adam Mickiewicz University, Św. Marcin 90, 61-809 Poznań, Poland.

[2]Department of Animal Taxonomy and Ecology, Adam Mickiewicz University, Umultowska 89, 61-614 Poznań, Poland

[*]corresponding author, katarzyna.fratczak@amu.edu.pl

Content

Fig. 1. Differences between males and females of *C. maxillosus* in duration of life stages at 15°C, 17.5°C, 20°C, 22.5°C, 25°C, 27.5°C, 30°C; ■ – mean, whiskers – standard error of the mean; E – the egg stage, L1 – first larval stage, L2 – second larval stage, L3 – third larval stage, P – pupal stage.

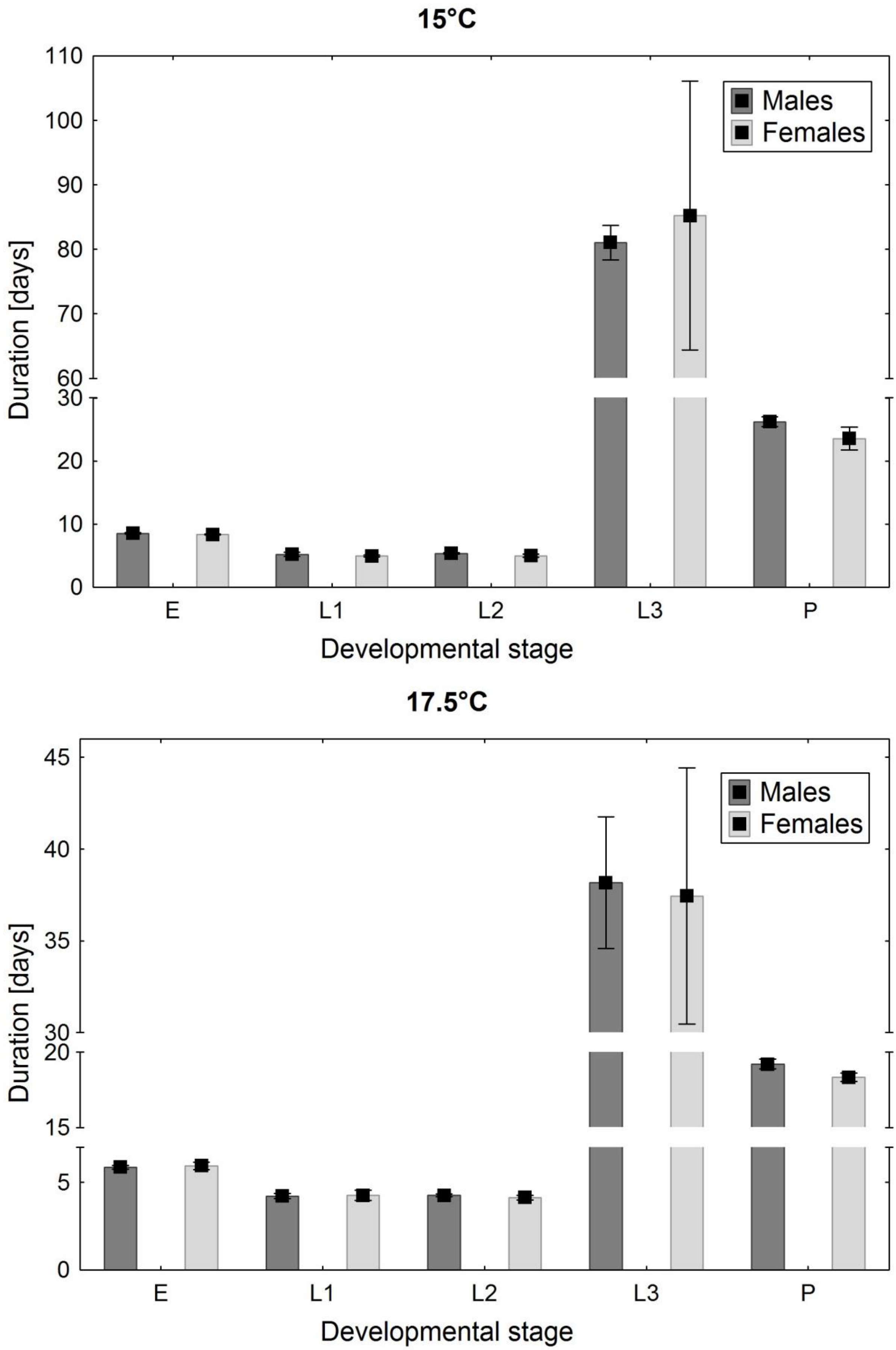

15°C
Duration [days]
110
100
90
80
70
60
30
20
10
0
Males
Females
E
L1
L2
L3
P
Developmental stage
17.5°C
Duration [days]
45
40
35
30
20
15
5
0
Males
Females
E
L1
L2
L3
P
Developmental stage

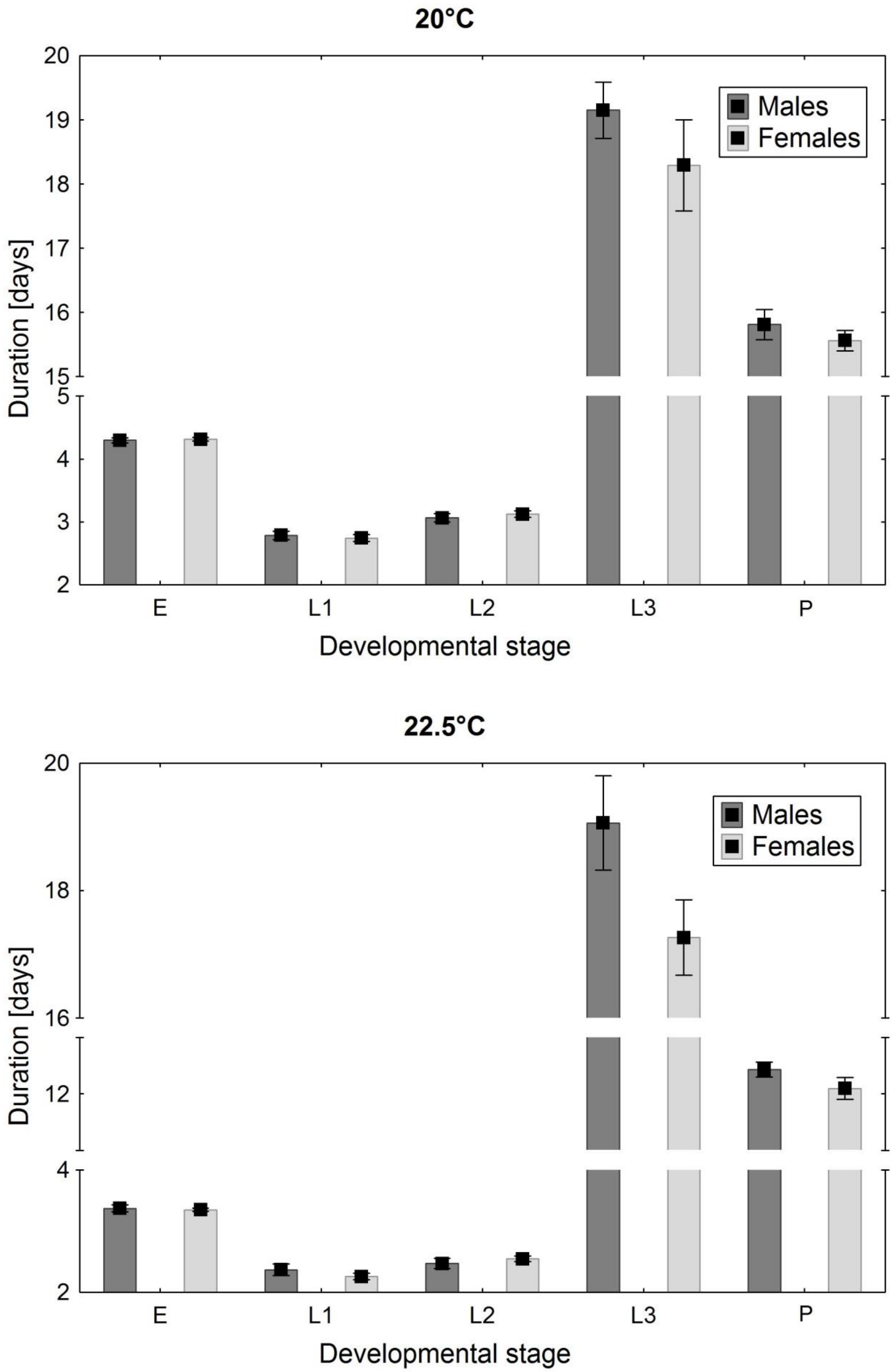
20°C
Duration [days]
20
19
18
17
16
15
5
4
3
2
Males
Females
E
L1
L2
L3
P
Developmental stage
22.5°C
Duration [days]
20
18
16
12
4
2
Males
Females
E
L1
L2
L3
P
Developmental stage

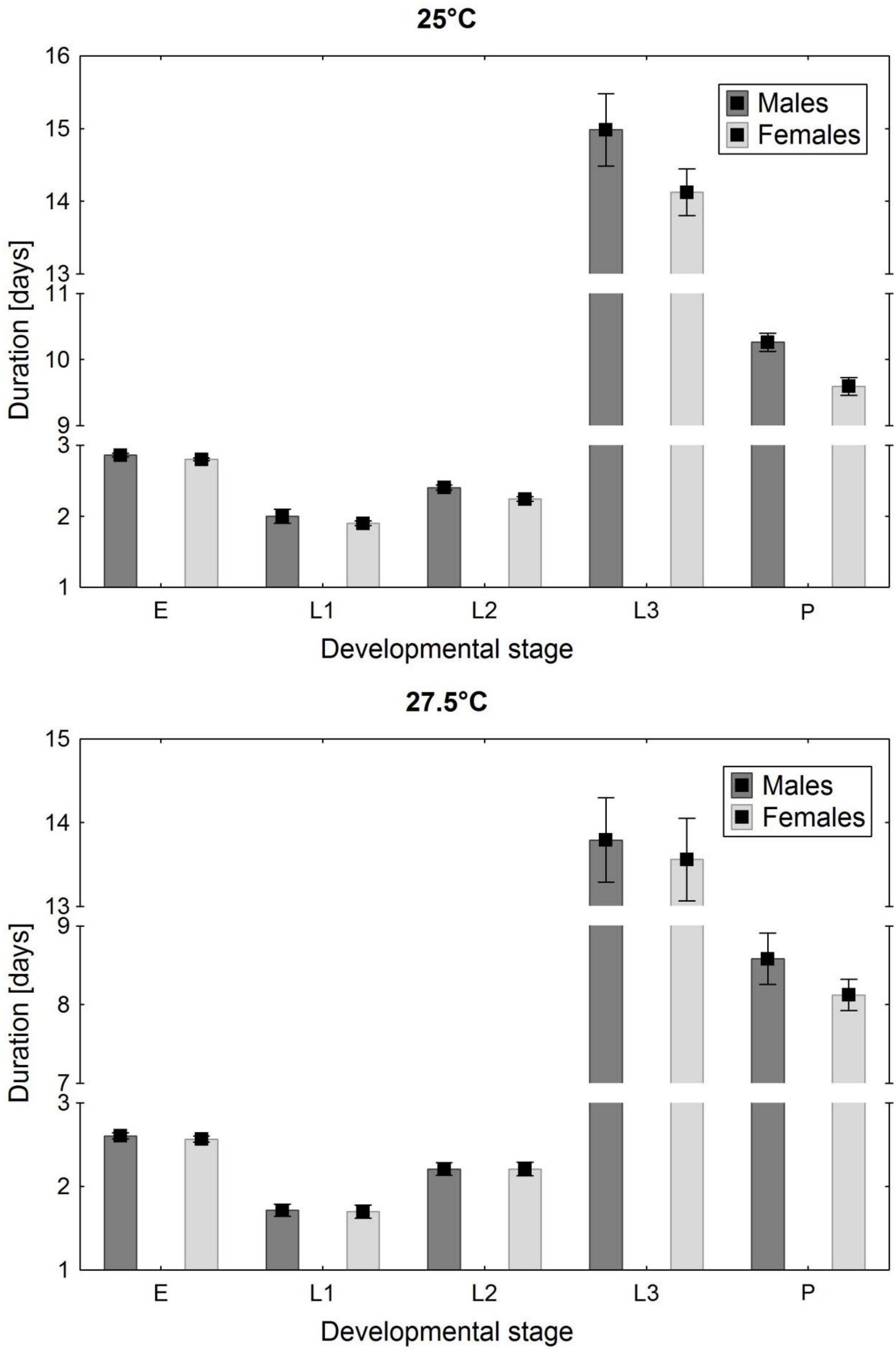
25°C
16
15
14
13
11
10
9
3
2
1
Males
Females
Duration [days]
E
L1
L2
L3
P
Developmental stage
27.5°C
15
14
13
9
8
7
3
2
1
Males
Females
Duration [days]
E
L1
L2
L3
P
Developmental stage

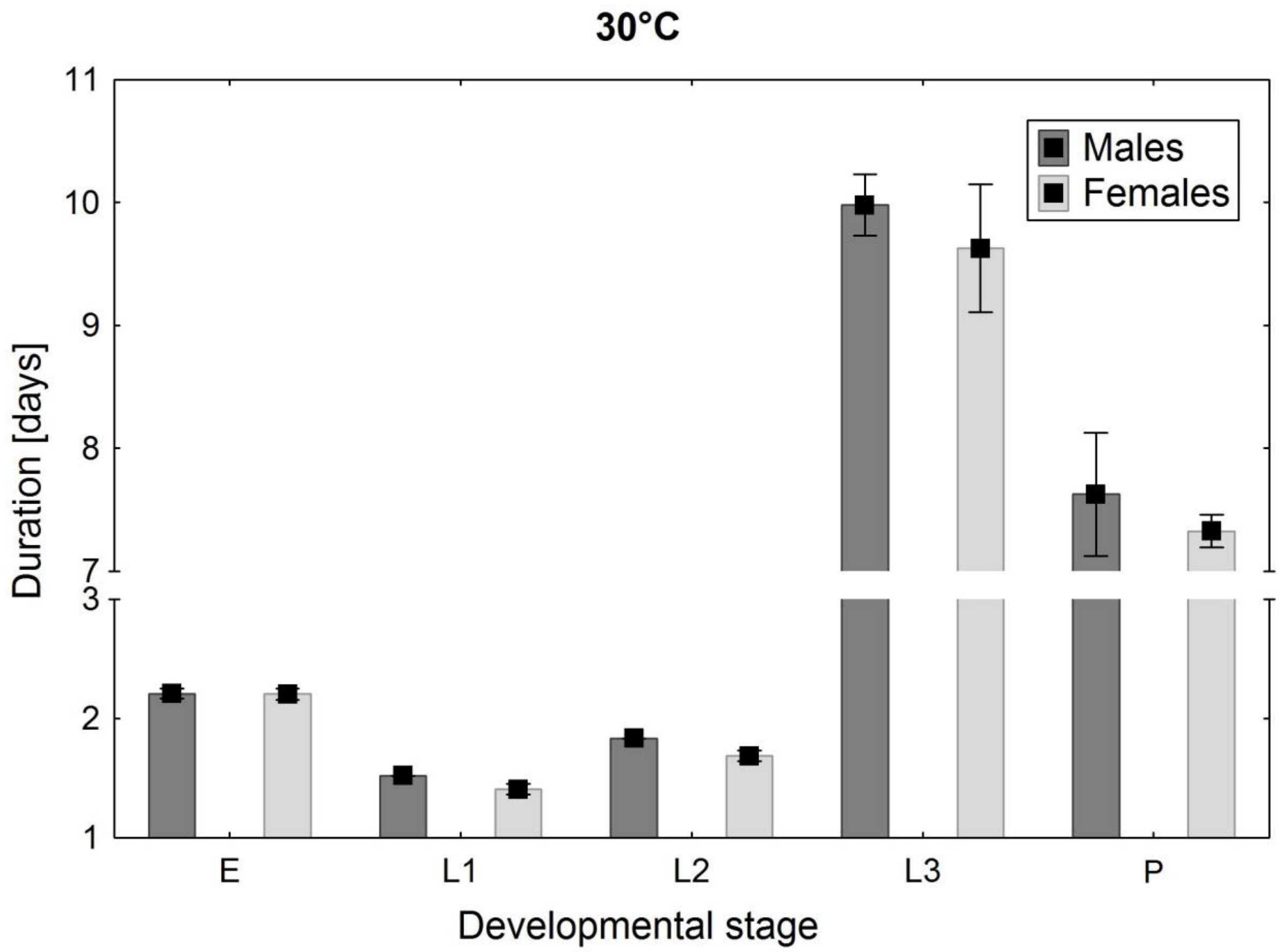

Fig. 1. Differences between males and females of *C. maxillosus* in duration of life stages at 15°C, 17.5°C, 20°C, 22.5°C, 25°C, 27.5°C, 30°C; ■ – mean, whiskers – standard error of the mean; E – the egg stage, L1 – first larval stage, L2 – second larval stage, L3 – third larval stage, P – pupal stage.
